# Meningovascular Inflammation in Cerebral Amyloid Angiopathy‐Related Cortical Superficial Siderosis

**DOI:** 10.1002/acn3.70315

**Published:** 2026-01-14

**Authors:** Philipp Arndt, Eya Khadhraoui, Sebastian J. Müller, Katja Neumann, Hendrik Mattern, Sven G. Meuth, Valentina Perosa, Andreas Charidimou, Stefanie Schreiber

**Affiliations:** ^1^ Department of Neurology Otto‐Von‐Guericke University Magdeburg Germany; ^2^ German Center for Neurodegenerative Diseases (DZNE) Within the Helmholtz Association Magdeburg Germany; ^3^ Department of Neuroradiology Otto‐Von‐Guericke University Magdeburg Germany; ^4^ Biomedical Magnetic Resonance, Faculty of Natural Sciences Otto‐Von‐Guericke University Magdeburg Germany; ^5^ Center of Behavioral Brain Sciences, CBBS Magdeburg Germany; ^6^ Department of Neurology Heinrich‐Heine‐University Düsseldorf Germany; ^7^ J. Philip Kistler Stroke Research Center, Department of Neurology Massachusetts General Hospital, Harvard Medical School Boston Massachusetts USA; ^8^ Department of Neurology Boston University Chobanian & Avedisian School of Medicine Boston Massachusetts USA

**Keywords:** cerebral amyloid angiopathy, cortical superficial siderosis, inflammation, vessel wall imaging

## Abstract

The role of inflammation in cortical superficial siderosis (cSS), a marker of cerebral amyloid angiopathy (CAA) linked to high hemorrhage risk, is unclear. We examined 15 patients with cSS using 3 T post‐contrast vessel wall MRI (VWI) and CSF analysis. Although only 27% met current CAA‐ri criteria, 93% showed vessel wall enhancement or sulcal hyperintensities near cSS, frequently extending beyond. Seven patients with follow‐up VWI demonstrated corticosteroid‐responsive regression of inflammation. CSF albumin quotients, indicating blood–brain barrier dysfunction, correlated with MRI inflammation scores. These findings reveal subclinical meningovascular inflammation in cSS and support VWI for detecting a broader CAA‐related inflammation spectrum.

## 1 Background

Sporadic cerebral amyloid angiopathy (CAA) is typically an age‐related, slowly progressive, and largely non‐inflammatory small vessel disease, characterized by the deposition of beta‐amyloid (Aβ) within the walls of cortical and leptomeningeal vessels [1]. CAA is now well established as a common cause of lobar intracerebral hemorrhage (ICH) and a major contributor to cognitive impairment in older individuals. In a subset of patients, however, preexisting CAA may rarely trigger an autoinflammatory response directed against vascular Aβ, resulting in inflammatory variants of the disease [2]. This condition, known as CAA‐related inflammation (CAA‐ri) in its typical form, presents with a distinct clinical and radiological phenotype, often marked by acute or subacute neurological symptoms and imaging findings that diverge from the typical profile of sporadic CAA. According to current diagnostic criteria, a diagnosis of CAA‐ri requires progressive neurological symptoms accompanied by confluent, often asymmetric, white matter hyperintensities extending into the subcortical white matter on MRI [3].

However, recently the concept of CAA‐ri spectrum disorders was introduced to acknowledge a broader continuum of neuroinflammatory involvement in sporadic CAA [2]. This emerging framework suggests that neuroinflammation may play an underrecognized role in cases of seemingly sporadic (i.e., non‐inflammatory) CAA and offers a more inclusive toolkit to diagnose CAA‐ri spectrum disorders. The concept was grounded on a retrospective case series of six patients with cortical superficial siderosis (cSS) and a clinical presentation of prolonged, unremitting CAA‐related transient focal neurological episodes (TFNEs), as well as other cases increasingly encountered in CAA clinical practice, who do not meet the current diagnostic criteria for the typical CAA‐ri syndrome [4, 5]. While these patients failed to fulfill established, but arguably limited in scope, CAA‐ri diagnostic criteria, they nonetheless exhibited inflammatory features on imaging—including sulcal, leptomeningeal, cortical vessel wall, and parenchymal enhancement on contrast‐enhanced and vessel wall MRI—suggestive of underlying blood–brain barrier (BBB) dysfunction and neuroinflammation [4].

We present a well characterized series of 15 patients with CAA‐related cSS, encompassing a range of CAA‐related clinical phenotypes and available cerebrospinal fluid (CSF) data who underwent post‐contrast MRI with high‐resolution vessel wall imaging (VWI). This series provides further evidence for a cSS‐linked, widespread neuroinflammatory process across diverse clinical presentations, lending additional support and validity to the emerging concept of CAA‐ri spectrum disorders.

## 2 Methods

### 2.1 Patients

This retrospective, consecutive case series includes 15 patients with CAA–related cSS and associated clinical symptoms who underwent VWI and post‐contrast MRI at the Department of Neurology, Otto‐von‐Guericke University Magdeburg. Between May 2022 and September 2025, 81 patients with probable CAA (Boston criteria version 2.0) were identified in the Magdeburg CAA database. Fifteen of these patients had cSS and received VWI as part of their diagnostic workup and were therefore included in the present analysis. Baseline characteristics (e.g., age, sex, and prevalence of lobar ICH) did not differ between included and excluded patients (all *p* > 0.05). The study was approved by the local ethics committee (No. 331 07/2017; addendum 11/2021).

### 2.2 MRI Data

Patients underwent a standardized MRI (median 5 days after clinical presentation, range: 0–8 days), including T1‐weighted, T2‐weighted, FLAIR, T2*‐weighted sequences (gradient‐recalled echo), diffusion‐weighted imaging, and pre‐ and post‐contrast VWI (black blood imaging). High‐resolution VWI was acquired after administration of Gadovist using a transversal, single‐slab, non‐selective 3D turbo spin echo sequence with generalized autocalibration partial parallel acquisition for image acceleration and spectral attenuated inversion recovery for fat suppression. Whole‐brain coverage was achieved with a 200 mm field of view, isotropic voxel size of 0.8 mm, repetition time of 700 ms, and echo time of 23 ms. All scans were performed on a 3‐Tesla MRI system (Philips Achieva, Best, Netherlands). In the three ICH and two acute subarachnoid hemorrhage (SAH) cases, MRI was performed a median of 7 days after admission (range 1–8).

Two experienced senior neuroradiologists (E.K. and S.M.) independently analyzed all sequences. The Auriel criteria were applied for the assessment of CAA‐ri [3]. Vascular lesions were classified according to the STRIVE‐2 criteria [6]. The Fazekas score was used to quantify white matter hyperintensities (WMH) [7]. The global cortical atrophy scale to assess atrophy [8], and the Microbleed Anatomical Rating Scale to evaluate cerebral microbleeds (CMB) [9]. VWI was assessed separately for large vessels (A1–A3, M1–M3, P1–P3, vertebral arteries, basilar artery), medium‐sized leptomeningeal vessels, and small cortical vessels. The number of contrast‐enhancing vessel walls was counted. All enhancement findings were cross‐checked with pre‐contrast VWI sequences to minimize artifacts. If uncertainty remained regarding whether an enhancing structure was arterial, it was not classified as vessel wall enhancement. Veins were differentiated based on their typical anatomical trajectory, continuity, and morphology. Sulcal hyperintensities or inflammatory parenchymal edema was evaluated on native FLAIR images. Since sulcal hyperintensities may also reflect (sub)acute convex subarachnoid hemorrhage, corresponding signal abnormalities were carefully checked on native T1‐weighted images.

Interrater reliability was assessed for the presence of leptomeningeal enhancement and for the quantitative assessment of enhancing vessels in all available scans. Agreement for binary variables was evaluated using Cohen's κ, and for quantitative variables using the intraclass correlation coefficient (ICC, two‐way random‐effects model, absolute agreement). Consensus was reached by joint re‐evaluation of the images. Agreement for the presence of leptomeningeal enhancement was substantial (Cohen's *κ* = 0.74). Quantitative assessments showed good agreement for the number of leptomeningeal enhancing vessels (ICC = 0.70, 95% CI 0.29–0.88) and moderate agreement for the number of cortical enhancing vessels (ICC = 0.60, 95% CI 0.28–0.80).

To generate a composite score for inflammation, we propose to sum the following results: leptomeningeal and cortical vessel wall enhancement on VWI, and sulcal hyperintensities and inflammatory parenchymal edema on native FLAIR (for each feature: presence—1 point; no—0 points). This resulted in a composite MRI inflammation score ranging from 0 to 4.

### 2.3 Cerebrospinal Fluid

Lumbar puncture was performed as part of the diagnostic workup. One ICH patient underwent lumbar puncture 45 days after initial admission, and two SAH patients had lumbar puncture 1 and 3 days after initial admission. CSF samples were centrifuged at 4°C, aliquoted, and stored at −80°C. Biomarkers including Aβ_42/40_ ratio, phosphorylated Tau 181 (pTau), total Tau (tTau), and neurofilament light (NfL) were measured using automated immunoassays (LUMIPULSE G600 II, Fujirebio). The ATN nomenclature refers to CSF markers of amyloid pathology (A), tau phosphorylation (T), and neurodegeneration (N). The thresholds applied in this study (Aβ_42/40_ < 0.69, pTau > 70 pg/mL, tTau > 404 pg/mL) follow established cut‐offs for Alzheimer's disease biomarker classification but not diagnostic criteria for CAA [10]. CSF and serum albumin and IgG were analyzed by rate nephelometry (Immage 800, Beckman Coulter). The albumin quotient (×10^−3^; Qalb) assessed BBB dysfunction, and the IgG index evaluated intrathecal IgG synthesis [11].

### 2.4 Statistical Analysis

Continuous variables are presented as median (range), and categorical variables as proportions. Qalb, a CSF marker of BBB dysfunction, was correlated with the composite MRI inflammation score using linear regression. Analyses were performed with GraphPad Prism 10.0.3.

## 3 Results

Demographic, clinical and imaging characteristics of the included patients are summarized in Table 1. The median age was 78 years (range: 66–91 years), and eight patients (53%) were female. All patients exhibited cSS, with 73% showing a disseminated pattern (≥ 4 sulci affected) and 27% a focal pattern (≤ 3 sulci). Four patients met the current diagnostic criteria for CAA‐ri [3]. Of note, 14 (93%) patients fulfilled the recently suggested criteria for CAA‐ri spectrum disorders. Clinical presentations included (sub)acute focal neurological deficits due to ICH, SAH, or vasogenic edema, progressive cognitive decline, TFNE, in one patient transient visual symptoms that justified MRI, and in one patient a focal neurological deficit without an acute lesion (Table 1).

**TABLE 1:** Characteristic of patients with cortical superficial siderosis who meet vs. do not meet current criteria for CAA‐related inflammation.

	All patients *n* = 15	Not meeting clinical and imaging CAA‐ri criteria *n* = 11	Meeting the clinical and imaging CAA‐ri criteria *n* = 4
Age	78 (66–91)	78 (66–91)	78 (69–88)
Female	8 (53%)	7 (64%)	1 (25%)
*Clinical presentation*			
Progressive cognitive decline	3 (20%)	3 (27%)	0 (0%)
Lobar ICH	3 (20%)	3 (27%)	0 (0%)
Focal motor deficit and SAH	2 (13%)	2 (18%)	0 (0%)
TFNE	2 (13%)	1 (9%)	1 (25%)
Focal neurological deficit with vasogenic edema	2 (13%)	0 (0%)	2 (50%)
Transient visual symptoms	1 (7%)	0 (0%)	1 (25%)
Focal neurological deficit without acute lesion	1 (7%)	1 (9%)	0 (0%)
Seizure and SAH	1 (7%)	1 (9%)	0 (0%)
*CAA markers on MRI*			
Disseminated cSS	11 (73%)	9 (82%)	2 (50%)
Number of lobar CMB	5 (0–400)	7 (0–23)	10 (0–400)
Severe CSO PVS (> 20)	15 (100%)	11 (100%)	4 (100%)
Multispot WMH pattern	13 (87%)	10 (91%)	3 (75%)
*Other CSVD markers on MRI*			
WMH Fazeka scale [0–6]	5 (2–6)	5 (3–6)	4.5 (2–6)
Severe BG PVS (> 20)	7 (47%)	5 (45%)	2 (66%)
Presence of lacune	9 (60%)	6 (55%)	3 (75%)
Global cortical atrophy score	2 (1–3)	2 (1–2)	2 (1–3)
*Cerebrospinal fluid*	*n* = 11	*n* = 8	*n* = 3^a^
A + T − N−	4 (36%)	3 (38%)	1 (33%)
A + T − N+	4 (36%)	3 (38%)	1 (33%)
A + T + N+	3 (27%)	2 (25%)	1 (33%)
Neurofilament light [pg/mL]	1866 (895–13,619)	1602 (895–7,919)	2968 (2017–13,619)
Protein [g/L]	0.50 (0.37–1.31)	0.50 (0.37–1.31)	0.50 (0.49–0.52)
Albumin quotient	10.3 (5.3–14.0)	9.5 (5.3–14.0)	10.9 (8.3–13.0)
IgG‐index	0.50 (0.45–0.70)	0.50 (0.45–0.60)	0.70 (0.50–0.70)

*Note:* Data are presented as median (range) or as proportions.

Abbreviations: BG, basal ganglia; CAA‐ri, cerebral amyloid angiopathy related inflammation; CMB, cerebral microbleed; CSO, centrum semiovale; cSS, cortical superficial siderosis; CSVD, cerebral small vessel disease; ICH, intracerebral hemorrhage; MRI, magnetic resonance imaging; PVS, perivascular spaces; SAH, subarachnoid hemorrhage; TFNE, transient focal neurological episodes; WMH, white matter hyperintensities.

^a^ One case underwent lumbar puncture and CSF analysis after anti‐inflammatory treatment (protein 0.49 g/L, albumin quotient 8.3, IgG‐index 0.70).

**FIGURE 1:**
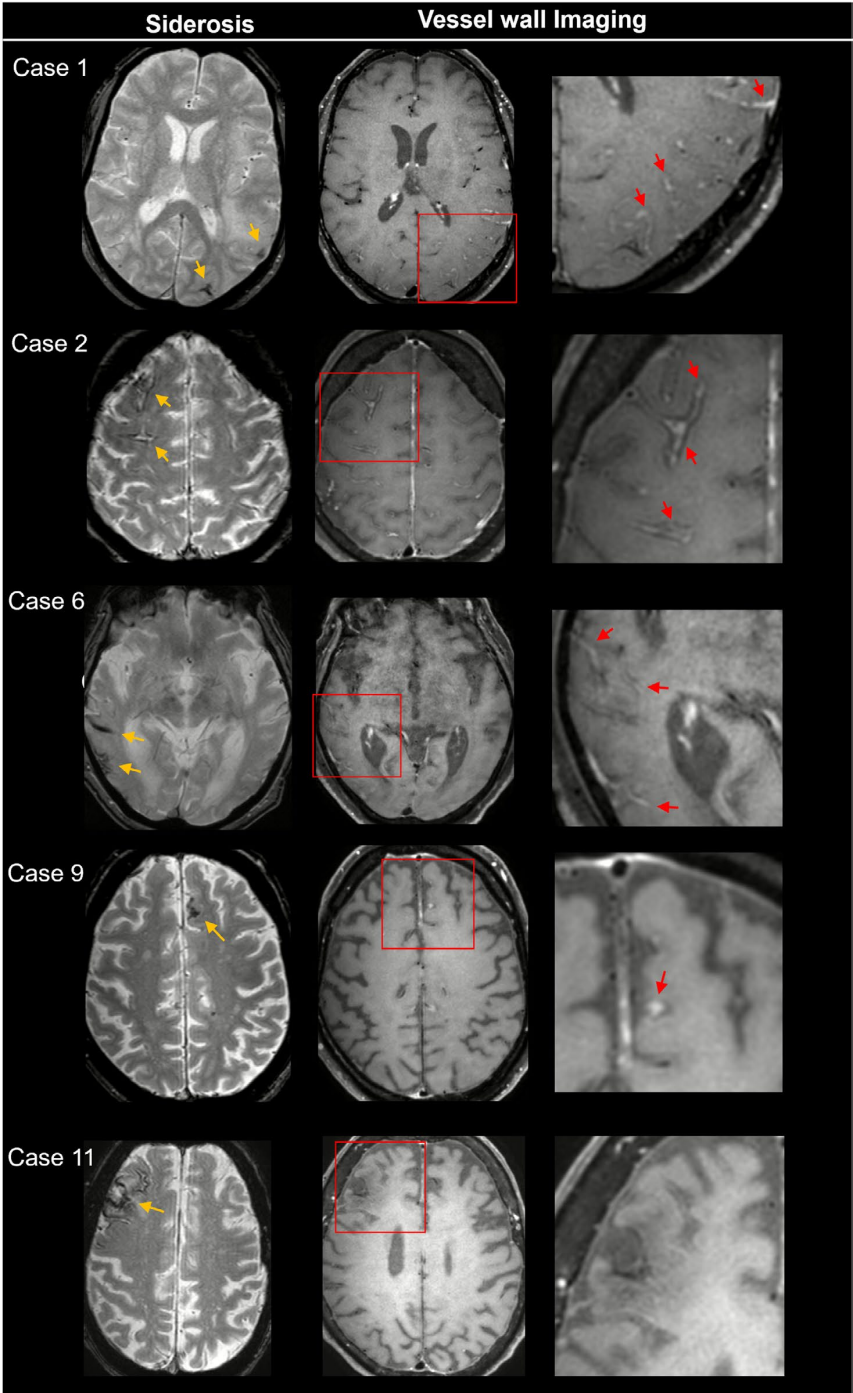
Anatomical overlap of cortical superficial siderosis and vessel wall enhancement on brain MRI. Five representative cases illustrate the variability in the severity of vessel wall enhancement on post‐contrast vessel wall imaging. Cases 1 and 2 demonstrate severe enhancement with typical “donut‐signs”, cases 6 and 9 show moderate enhancement, and case 11 shows no detectable vessel wall enhancement. Corresponding clinical and imaging variables for each patient are demonstrated in Tables 1 and 2. Image interpretation was guided by pre‐contrast sequences and anatomical landmarks to avoid venous misclassification.

**FIGURE 2:**
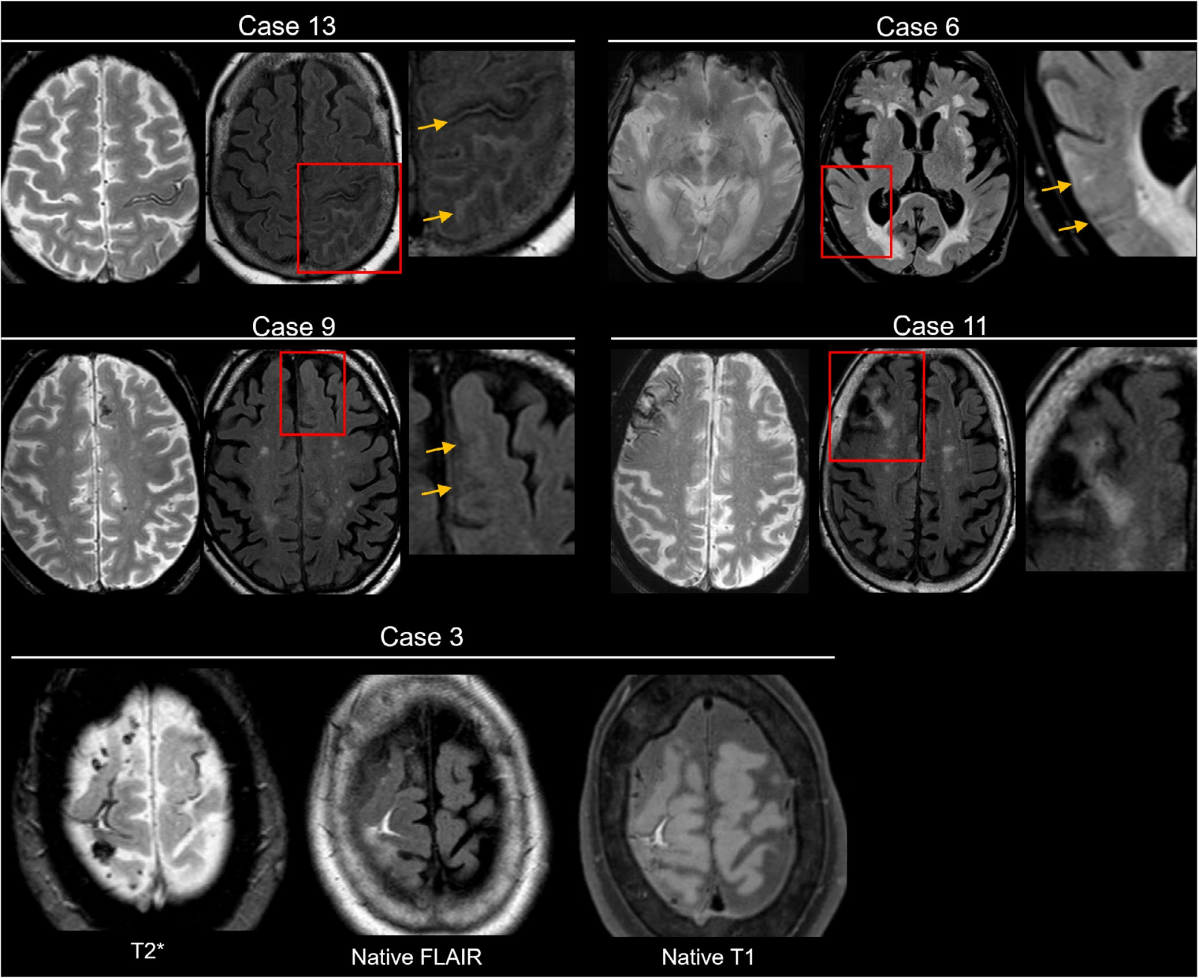
Anatomical overlap of cortical superficial siderosis and sulcal hyperintensities on native FLAIR. Five representative cases illustrate the variability in sulcal hyperintensities' extent on native FLAIR sequences. Case 13 shows a severely affected patient meeting current clinical and radiological CAA‐ri criteria. Cases 6 and 9 display localized sulcal hyperintensities that correspond to the side of siderosis, while case 11 shows no detectable sulcal hyperintensities. Of note, sulcal hyperintensities may also reflect (sub)acute subarachnoid hemorrhage, as in case 3, where corresponding signal abnormalities are also visible on native T1‐weighted sequences. Clinical and imaging variables for each case are summarized in Tables 1 and 2.

The majority of patients showed neuroimaging evidence of inflammation on VWI, including leptomeningeal vessel enhancement (*n* = 14 patients, 93%) or cortical vessel wall enhancement (*n* = 13 patients, 87%), while inflammatory parenchymal (vasogenic) edema was less frequent (*n* = 3 patients, 20%) (Table 2). Among those with vessel wall enhancement, sulcal hyperintensities were visible on native FLAIR in all 14 patients, without corresponding hemorrhage on T1‐weighted MRI. Both vessel wall and sulcal enhancement were pronounced in areas affected by cSS, although vessel wall enhancement was also observed distant from cSS (Figures 1 and 2). Notably, ten patients exhibited diffuse vessel wall enhancement throughout the brain. Enhancement of large‐caliber arteries was seen in nine patients (60%). Conversely, one case with disseminated cSS (case 11) did not show an inflammatory process on VWI or native FLAIR MRI. In all included patients, VWI MRI was performed prior to the initiation of corticosteroid therapy.

**TABLE 2:** MRI markers of inflammation.

Case	Cortical superficial siderosis	Parenchymal (vasogenic) edema	Sulcal HI on native FLAIR	LME	Counts of LM VWE	Counts of cortical VWE	Large VWE	Treatment for inflammation
*Cases not meeting the CAA‐ri criteria*								
1	Bilateral, disseminated	No	Yes	Bilateral, diffuse	64	5	Right M3	Methylprednisolone 0.5 g/day iv for 3 days
2	Right, disseminated	No	Yes	Bilateral, diffuse	61	5	Bilateral M2	No
3	Bilateral, disseminated	No	Yes	Bilateral, diffuse	63	6	No	Methylprednisolone 1 g/day iv for 3 days
4	Bilateral, disseminated	No	Yes	Bilateral, diffuse	52	9	No	Methylprednisolone 1 g/day iv for 3 days
5	Bilateral, disseminated	No	Yes	Bilateral, diffuse	46	15	Right M3	No
6	Bilateral, disseminated	No	Yes	Bilateral, parieto‐temporal	41	2	Right M1 & P1	Methylprednisolone 1 g/day iv for 3 days
7	Bilateral, disseminated	No	Yes	Bilateral, diffuse	40	8	Bilateral M2 and M3	Methylprednisolone 1 g/day iv for 5 days
8	Bilateral, disseminated	No	Yes	Bilateral, diffuse	33	7	Bilateral M2 and left P2	No
9	Bilateral, disseminated	Left, parietal	Yes	Bilateral, fronto‐parietal	12	4	Left M1, right M2	Methylprednisolone 1 g/day iv for 3 days
10	Left, focal	No	Yes	Bilateral, temporo‐occipital	15	0	No	Methylprednisolone 1 g/day iv for 3 days
11	Right, disseminated	No	No	No	0	0	No	Methylprednisolone 1 g/day iv for 3 days
*Cases meeting the CAA‐ri criteria*								
12	Left, focal	Left, parietal	Yes	Bilateral, diffuse	56	7	Bilateral M2 and left P1	Methylprednisolone 1 g/day iv for 3 days
13	Left, focal	No	Yes	Bilateral, diffuse	46	5	No	Methylprednisolone 1 g/day iv for 5 days
14	Bilateral, disseminated	No	Yes	Bilateral, diffuse	35	4	Bilateral M2	Methylprednisolone 1 g/day iv for 3 days
15	Right, disseminated	Right, frontal	Yes	Right, fronto‐temporal	19	2	No	Methylprednisolone 0.5 g/day iv for 5 days

Abbreviations: CAA‐ri, cerebral amyloid angiopathy related inflammation; d, days; g, gram; HI, hyperintensities; iv, intravenous; LME, leptomeningeal enhancement; MRI, magnetic resonance imaging; VWE, vessel wall enhancement.

One of three patients with acute ICH received high‐dose corticosteroid therapy, though treatment was initiated during a second hospitalization 45 days later. All other patients were offered corticosteroids during the index admission. Treatment regimens consisted of 0.5 or 1 g intravenous methylprednisolone per day for 3 or 5 days. Detailed treatment information for each case is provided in Table 2. All seven patients with neuroimaging evidence of inflammation on VWI, who received corticosteroid treatment and had follow‐up MRI (median follow‐up 6 months, range 2–25), demonstrated a reduction in vessel wall enhancement and parenchymal edema. One patient (case 13) initially demonstrated radiological improvement but subsequently developed recurrent clinical symptoms and renewed inflammatory changes on VWI despite corticosteroid treatment; a second course of corticosteroids was therefore initiated.

CSF samples were available for eleven patients, ten of whom were obtained prior to initiation of anti‐inflammatory treatment. All patients were within the Alzheimer's disease continuum (A+T−N− *n* = 4, A+T−N+ *n* = 4, A+T+N+ *n* = 3). Elevated total protein levels and increased Qalb values were consistent with BBB dysfunction (Table 1). Notably, Qalb demonstrated a positive association with the MRI‐based inflammation score (*n* = 10, *R* ^2^ = 0.64, *p* = 0.006), even after exclusion of patients meeting CAA‐ri criteria (*n* = 8, *R* ^2^ = 0.63, *p* = 0.018).

All 15 patients were followed clinically and through repeated neuroimaging for a median of 3.1 months (range 1–25 months; total follow‐up 7.3 patient‐years). One patient with an initial ICH and vessel wall enhancement (case 2) experienced a recurrent lobar ICH after 2 months; corticosteroid therapy had not yet been initiated at that time.

## 4 Discussion

This case series provides further supports to the concept of CAA‐ri spectrum disorders [2], highlighting the presence of an inflammatory process with associated BBB dysfunction in patients with CAA‐related cSS, regardless of clinical phenotype. Using high‐resolution VWI and native FLAIR MRI, we identified vessel wall enhancement in close anatomical proximity to cSS in most patients, suggesting a spatial and potentially pathophysiological link between cSS and underlying meningovascular inflammation. Notably, we observed substantial variability in the extent and severity of inflammatory changes across patients, including both those who did and did not fulfill current diagnostic CAA‐ri criteria. One patient with cSS lacked neuroimaging evidence of inflammation entirely, underscoring the heterogeneity of presentations. Still, our findings provide a pragmatic imaging‐based approach to recognizing neuroinflammation in CAA, particularly in cases with cSS, and lay the groundwork for more refined pathophysiological models of CAA‐ri spectrum disorders. Ultimately, this framework may help guide the selective use of immunomodulatory therapy aimed at reducing CAA‐related vascular injury and bleeding, an unmet need in the field.

Importantly, eleven of fifteen patients in our series did not meet the current CAA‐ri diagnostic criteria, which remain anchored solely on the presence of confluent, asymmetric WMH on FLAIR MRI sequences [3]. Yet, ten of these eleven cases (93%) showed clear imaging evidence of meningovascular inflammation, an observation consistent with prior findings in a cSS‐TFNE case series, where five of six patients (83%) demonstrated inflammation on VWI despite not meeting formal CAA‐ri criteria [4]. These findings suggest that contrast‐based VWI offers sensitivity for detecting inflammation in CAA and supports the notion of their inclusion in the recently suggested framework of CAA‐ri spectrum disorders [2]. The revised framework must also better reflect the diversity of neuroinflammatory patterns across CAA presentations, particularly in patients with cSS, who may harbor clinically significant, yet under‐recognized, inflammation. In parallel, the integration of fluid biomarkers reflecting meningovascular inflammation, including indicators of endothelial activation, BBB dysfunction, or specific inflammatory signaling pathways, could enhance diagnostic precision and complement imaging‐based assessments. Together, these tools may help redefine the boundaries of CAA‐ri spectrum disorders, moving beyond the classical syndrome to recognize atypical, yet treatable, presentations. From a pathophysiological standpoint, cSS in CAA is thought to result from recurrent, self‐limiting leptomeningeal hemorrhages, leading to hemosiderin deposition along the superficial cortical layers [12]. These bleeds may result from the rupture of fragile, amyloid‐laden pial vessels. Individuals with CAA‐related cSS often show rapid disease progression and are at increased risk for future fatal ICH [13, 14]. Preliminary evidence, including from our cohort, suggests that corticosteroid therapy induces regression of vessel wall enhancement and inflammation [4, 15]. Whether meningovascular inflammation contributes to vessel wall fragility and bleeding susceptibility, and whether anti‐inflammatory strategies can modify long‐term outcomes, including survival and hemorrhage risk, warrants further investigation.

## 5 Limitations

This study is limited by its retrospective design, small sample size, and lack of pathological confirmation of vascular inflammation. These factors restrict generalizability and introduce potential selection bias. Nonetheless, the consistency of neuroimaging findings and supportive CSF data offer compelling indirect evidence for a clinically relevant meningovascular inflammatory process.

Time‐of‐flight MR angiography was not routinely acquired, limiting systematic correlation of VWI findings with vascular anatomy. While slow‐flowing veins can appear hyperintense, we aimed to exclude venous structures based on pre‐contrast sequences and typical morphology. Prior work in CNS vasculitis has shown that VWI can reliably detect inflammatory changes and differentiate them from non‐inflammatory vasculopathies when evaluated in conjunction with other MRI sequences [16, 17, 18]. Nevertheless, we acknowledge that the achievable spatial resolution increases with field strength, and early work at 7 T suggests that ultra‐high‐field VWI may improve the assessment of small‐vessel inflammatory involvement [19]. Contrast‐enhanced FLAIR sequences have also shown utility in detecting leptomeningeal enhancement in CAA, were associated with cSS volume and could be a practical alternative in settings with limited access to VWI protocols or lower field‐strength scanners [20, 21, 22]. However, unlike VWI, it does not permit direct visualization of mural vessel enhancement. Future prospective studies with larger cohorts are warranted to validate these observations and evaluate the effect of anti‐inflammatory treatment on clinical outcomes in patients with CAA‐related cSS.

## Author Contributions

S.S. contributed to the conception and design of the study; all the authors contributed to the acquisition and analysis of data; P.A., S.S., and A.C. contributed to drafting the text; P.A., E.K., and S.J.M. contributed to preparing the figures. All the authors contributed to a critical review of the manuscript.

## Funding

This study was supported by the BB‐DARS project, funded by the Deutsche Alzheimer Gesellschaft (DAlzG) and the Förderstiftung Dierichs.

## Conflicts of Interest

The authors declare no conflicts of interest.

## Data Availability

The corresponding author has full access to the data used in this manuscript and all data are available on reasonable request.
